# Is it always COVID-19 in acute febrile illness in the tropics during the pandemic?

**DOI:** 10.1371/journal.pntd.0010891

**Published:** 2022-11-02

**Authors:** Ayush Gupta, Farha Siddiqui, Shashank Purwar, Rajnish Joshi, Chiranjay Mukhopadhyay

**Affiliations:** 1 Department of Microbiology, All India Institute of Medical Sciences, Bhopal, Madhya Pradesh, India; 2 Department of General Medicine, All India Institute of Medical Sciences, Bhopal, Madhya Pradesh, India; 3 Department of Microbiology, Kasturba Medical College, Manipal Academy of Higher Education, Manipal, Karnataka, India; 4 Center for Emerging and Tropical Diseases, Manipal Academy of Higher Education, Manipal, Karnataka, India; University of North Carolina at Chapel Hill, UNITED STATES

## Introduction

Diagnosing cases of acute febrile illness (AFI) is often challenging for physicians in the tropics, wherein there is a long list of infectious diseases as differential diagnoses. Notable among them are malaria, dengue, typhoid, leptospirosis, and rickettsial infections depending upon the region and the seasonality [1]. Each of them requires specific management for treatment and possesses a definite risk of mortality, if not diagnosed early [2]. The risk of mortality increases further if the patient has underlying noncommunicable diseases notably diabetes, hypertension, and chronic kidney diseases among others. Since the beginning of 2020, Coronavirus Disease 2019 (COVID-19) has been an important differential diagnosis in patients presenting with AFI with or without respiratory complaints [3]. The clinical management further becomes complicated considering that there are high rates of coinfections with COVID-19 in tropical countries and such patients with coinfections also have a high risk of mortality [4]. Herein, we present 2 patients to highlight the fact that in the tropics, causes other than COVID-19, should be aggressively looked for in patients presenting with AFI. Furthermore, in the appropriate clinical setting and particularly in patients presenting with severe illness, coinfections should also be searched and ruled out.

## Case presentation

### Patient 1

A 25-year-old male with diabetes mellitus (DM) type 1, arrived at the emergency department (ED) with complaints of fever and dyspnea for 3 days. On examination, he was febrile, tachycardic, tachypneic, and in hypotensive shock. The patient’s random blood sugar (RBS) was 341 mg/dl and his initial complete blood count (CBC) was notable for a raised white blood cell count of 13,800 /μL with neutrophilic predominance. Erythrocyte sedimentation rate (ESR) and C-reactive protein (CRP) were 16 mm/h and 78.6 mg/L, respectively. The chest x-ray (CXR) was remarkable for increased broncho-vascular markings and the rest of the findings were normal. In view of shock, the patient was admitted to the intensive care unit (ICU) and was started on mechanical ventilation, piperacillin-tazobactam as empirical antibiotic therapy along with inotropes, fluids, and insulin. Various clinical samples were collected to rule out etiological causes of AFI prevalent in Indian settings, such as typhoid fever (blood culture and Widal test), malaria (rapid antigen detection tests), dengue (NS1 antigen detection test), HIV (serology), and urine culture (urinary tract infection), all of which tested negative except for blood culture. Simultaneously, in view of prominent broncho-vascular markings, nasopharyngeal and throat swabs were sent for real-time reverse transcriptase-polymerase chain reaction (rRT-PCR) to rule out infection by Severe Acute Respiratory Syndrome-Associated Coronavirus 2 (SARS-CoV-2). The patient tested positive for SARS-CoV-2 by the ICMR-NIV rRT-PCR confirmation assay (ICMR-NIV, Pune, India). On the second day, his blood cultures bottles (BCBs) (FA Plus, Bac-TAlert 3D, bioMerieux, Marcy l’Etoille, France) flagged positive and revealed the presence of gram-negative bacilli. Blood: broth mixture from BCB was subcultured onto the plated media namely Chocolate agar (CA), Blood Agar (BA), and MacConkey agar (MA) that were incubated aerobically. Within 48 hours of admission, the patient improved initially and his sugar levels were controlled with insulin therapy; however, on third day, the patient collapsed suddenly and died despite cardiopulmonary resuscitation.

**Fig 1:**
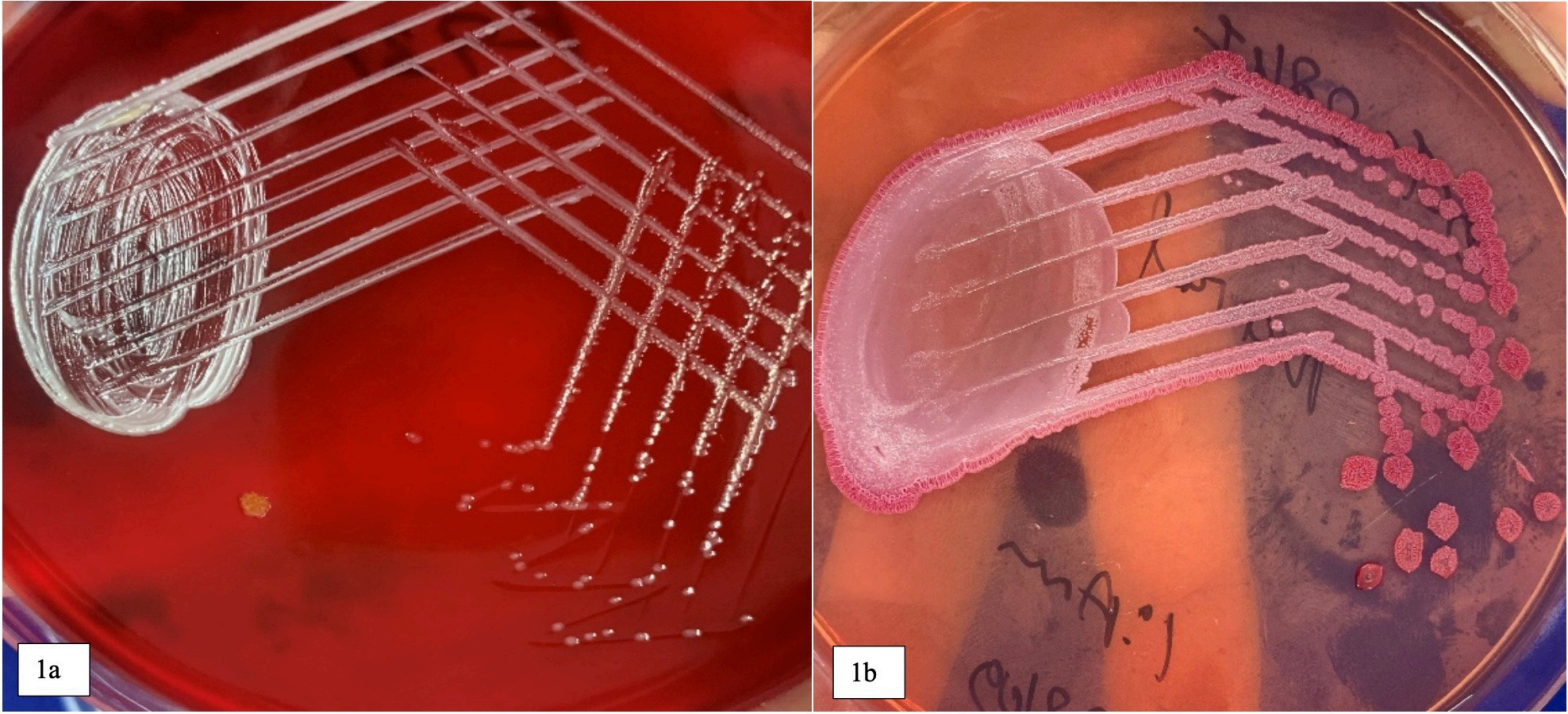
**(a)** Approximately 1–2 mm in size, white colored, round, smooth, elevated colonies with metallic sheen after 24 hours incubation on Sheep Blood agar. **(b)** Non-lactose fermenting, 2–4 mm in size, dry wrinkled colonies on MacConkey agar after 48–72 hours of incubation.

**Fig 2:**
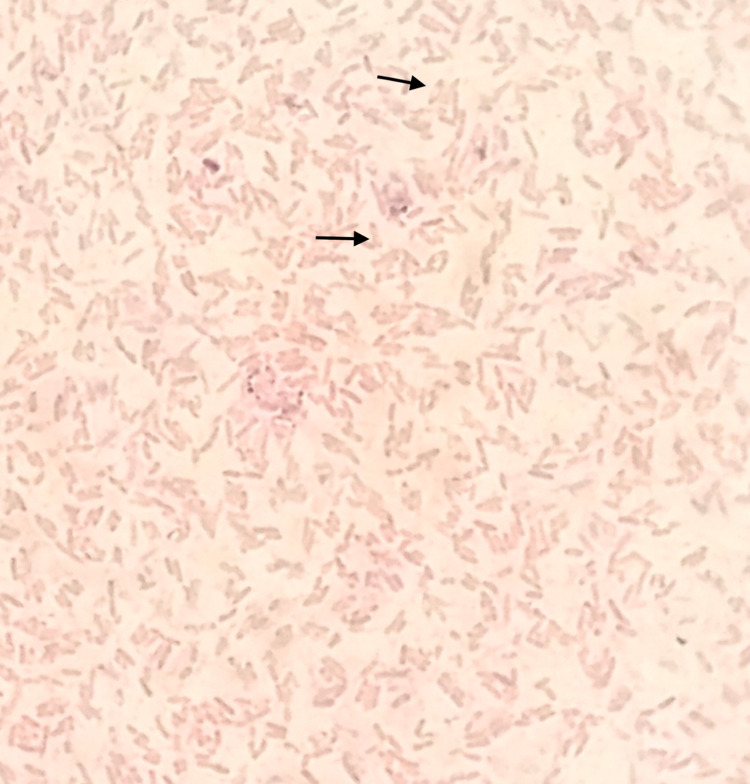
Gram staining showing gram-negative bacilli with safety-pin appearance.

On all plated media, small pin-point colonies were observed within 24 hours; however, they revealed their characteristics only after 48-hour incubation. The colonies were notable for being 1 to 2 mm in size, non-hemolytic, white in color, round in shape with metallic sheen on CA and BA (Fig 1A), and non-lactose fermenting on MA. On further incubation, the colonies became dark pink, dry, and wrinkled with radiating ridges on MA (Fig 1B). Gram staining from the colonies revealed gram-negative bacilli with bipolar staining (safety pin appearance) (Fig 2) and aroused the suspicion of *Burkholderia pseudomallei* considering the patient’s history of DM type 1 and clinical presentation of sepsis with pneumonia. The organism was motile by hanging drop examination, catalase positive and oxidase was only late positive. The isolate was identified as *B*. *pseudomallei* by VITEK-2 Compact (bioMerieux, Marcy l’Etoile, France) microbial identification and susceptibility testing system with a 99% probability of identification. The isolate was sent to the Center for Emerging and Tropical Diseases at Kasturba medical college, Manipal for confirming its identity. The isolate tested positive by monoclonal antibody-based latex agglutination test (Mahidol University, Bangkok, Thailand), Active Melioidosis detect lateral flow immunoassay (InBios International, Seattle, United States of America) and real-time PCR targeting T3SS1 gene, confirming its identity as *B*. *pseudomallei*. The isolate tested sensitive to ceftazidime, meropenem, doxycycline, and trimethoprim-sulphamethoxazole [5].

### Patient 2

A 58-year-old male patient, known diabetic (type 2) presented with high-grade fever and breathlessness in the ED. He was admitted previously to a hospital where he was being managed for diabetic ketoacidosis. Upon admission, he was tachypneic and tachycardic, oxygen saturation was 91%, and had bilateral crepitations on chest auscultation. CBC showed a WBC count of 4.73/μL with neutrophilic predominance; ESR and CRP were 24 mm/h and 260.5 mg/L, respectively. His CXR and computed tomography (CT) scan of the thorax showed patchy areas of consolidation with multiple discrete nodules in the bilateral lung parenchyma. The patient was suspected to be a case of COVID-19, however, tested negative for SARS-CoV-2 by rRT-PCR. The patient was started on meropenem and clindamycin empirically. On third day, blood cultures flagged positive and revealed the presence of gram-negative bacilli but by then, the patient had succumbed to cardiorespiratory failure. The isolate from blood culture was identified as *B*. *pseudomallei* by VITEK-2 Compact system, typical reactions on conventional biochemical tests and triple disk test (sensitive to amoxicillin-clavulanate and resistant to gentamicin and polymyxin B).

## Discussion

Melioidosis is a tropical illness caused by *B*. *pseudomallei*, a gram-negative bacterium commonly found in soil and freshwater. Infection in humans commonly occurs through inhalation, by direct inoculation on the skin due to trauma, and rarely ingestion. It is endemic throughout the tropics with Southeast Asia, Northern Australia and Indian subcontinent being the major hotspots. The disease exhibits seasonal variation with most cases being reported during the rainy season [6]. The clinical manifestations are protean, ranging from acute (defined as symptoms present for <2 months) to chronic illness (symptoms present for ≥2 months) involving a variety of organ systems [7]. The most common clinical presentation is community-acquired pneumonia (CAP) with or without sepsis, while skin and soft tissue involvement with abscess formation; bone and joint infection; genitourinary infection and visceral organ abscess involving the liver and spleen, are also notable [6,7]. Patients with DM are at significant risk for developing the disease, whereas hazardous alcohol use and chronic renal disease are other notable risk factors. The mortality rate associated with melioidosis range from 10% to 50% and is directly correlated with the diagnostic infrastructure available in the region [8]. It is essential to diagnose a case of melioidosis for the appropriate management as the treatment involves specific antimicrobials for a considerable duration. Treatment is given in 2 phases: intensive phase for 2 to 4 weeks by either ceftazidime, imipenem, or meropenem and eradication phase for 6 months by trimethoprim-sulphamethoxazole.

The 2 patients presented here resided in the state of Madhya Pradesh in central India. Previously, melioidosis has never been diagnosed within this region; however, it was diagnosed at other centers in patients belonging to this region [8–10]. One of these cases, the first reported case from India, was diagnosed in 1953, in a Scottish mining engineer who had stayed in the central province of India (under British rule) for 16 years. The patient was diagnosed with *Pfeifferella whitmori* (name later changed to *B*. *pseudomallei*) infection in Glasgow [9]. Two more cases attributed to central India were found in retrospective case reviews from institutes in south India, clinical details for which were not described individually [8,10]. At our center, we have diagnosed around 25 cases of melioidosis in the last 2 years despite the healthcare infrastructure being directed towards the management of the COVID-19 pandemic. Although patients are present throughout the year, there is an increase in the number of cases in the rainy season, i.e., from July to September. Both our patients presented during the rainy season in August and September months, respectively. Due to the severe nature of the illness, short duration of hospitalization and retrospective diagnosis after mortality, detailed history regarding exposure to soil and radiological investigations to assess visceral involvement could not be elicited. We believe that poor healthcare infrastructure coupled with a lack of awareness among clinicians may have contributed to the underreporting of melioidosis in this region for a long time.

A modeling study estimated around 165,000 cases worldwide with predicted case fatality in 89,000 cases (54%), a number considerably higher than diseases such as measles, dengue, and leptospirosis, which receive considerably more attention [11]. This study also predicted that the highest burden of melioidosis, around 44%, is in south Asia including India. Other regions of the world where the burden of melioidosis is high include Southeast Asia, Australia, sub-Saharan Africa, and Latin America. The burden of melioidosis in a country like India is masked by a lack of awareness among clinicians and microbiologists about the disease, under-developed microbiological facilities, and a lack of surveillance systems [11]. Few centers in India have the expertise to identify and diagnose cases of melioidosis. These centers are located in the coastal states in south India such as Karnataka, Kerala, and Tamil Nadu with adjoining union territory Puducherry and Odisha in eastern India [8,10,12–14]. However, it is now increasingly being recognized in other regions of the country including northern, north-western India [15,16], and now central India. Unlike countries in Southeast Asia like Thailand, melioidosis is not a notifiable disease in India under the Integrated Disease Surveillance Program (IDSP).

Our cases were diagnosed as these patients presented with bacteremia and blood samples grew the bacterium in pure cultures. Both patients presented with features of pneumonia and might have been diagnosed earlier if respiratory specimens had been taken early in the course of the illness. To increase the yield of *B*. *pseudomallei* from respiratory specimens, enrichment cultures are useful [6] but only a few centers in India employ them routinely [17,18]. Consequently, the proportion of melioidosis in CAP is grossly underestimated in India as only bacteraemic cases are likely to be diagnosed. Often, the bacterium is misidentified as *Pseudomonas aeruginosa* or *Acinetobacter* spp. due to a lack of awareness and expertise among microbiologists who often rely on widely prevalent conventional biochemical tests based on microbial identification (8). Automated microbial identification systems like VITEK-2 system can identify *B*. *pseudomallei* but fail to identify slow-growing isolates and sometimes misidentify them as *P*. *aeruginosa*, *P*. *fluorescens*, or *Sphingomonas paucomobilis*. Even mass spectrometry-based instruments, either VITEK MS (bioMerieux, Marcy l’Etoile, France) or Bruker Microflex Biotyper (Bruker Daltonic GmbH, Bremen, Germany) do not include *B*. *pseudomallei* in their standard database [6].

We presented 2 cases of AFI, which were initially suspected to be of COVID-19. Both patients suffered from DM and presented with severe illness and unfavorable clinical outcomes. Patient 1 turned out to be a case of coinfection of melioidosis with SARS-CoV-2. Recently, a case from the United States was reported in a patient of Vietnamese origin wherein COVID-19 led to the reactivation of melioidosis that ultimately proved fatal [19]. The purpose of presenting these cases of melioidosis is to remind a busy clinical community that in the tropics, melioidosis should always be kept in the differential diagnoses of CAP, particularly in diabetics as they may present with fulminant sepsis and ultimately prove fatal. Furthermore, cases of COVID-19 coinfections with other common causes of AFI in the tropics may present with aggressive clinical features.

To conclude, during the ongoing COVID-19 pandemic, clinicians must be vigilant regarding the possibility of other causes of AFI in tropical regions apart from COVID-19 and aggressively search for them. Patients with coinfections of other tropical illnesses and COVID-19 present with severe clinical manifestations and outcomes may be particularly poor. Melioidosis is increasingly being recognized in other regions of India with improvement in awareness among clinicians, microbiologists, and improvement in diagnostic facilities. There is an unmet need to develop a national surveillance system for melioidosis to further understand the epidemiology of this neglected tropical disease.

### Ethics statement

Waiver of consent from the Institutional Human Ethics Committee is taken as per letter no.: IHEC-LOP/2022/IL010 dated 2 March 2022.

**Key learning points**

- Melioidosis is an important differential in cases presenting with acute febrile illness in the tropics, especially in diabetics.
- Coinfections in COVID-19 should be aggressively searched for, especially in patients who present with severe clinical manifestations.
- Melioidosis is expanding its geographical footprint and is increasingly being recognized due to improvements in diagnostic facilities.
- Nationwide surveillance system needs to be established in India, which has the highest predicted burden of melioidosis in the world.
